# *PbCSE1* promotes lignification during stone cell development in pear (*Pyrus bretschneideri*) fruit

**DOI:** 10.1038/s41598-021-88825-0

**Published:** 2021-05-03

**Authors:** Jiahui Xu, Xingyu Tao, Zhihua Xie, Xin Gong, Kaijie Qi, Shaoling Zhang, Katsuhiro Shiratake, Shutian Tao

**Affiliations:** 1 Pear Engineering Research Centre, College of Horticulture, Nanjing Agricultural University, Nanjing, 210095 Jiangsu Province China; 2 State Key Laboratory of Crop Genetics and Germplasm Enhancement, Nanjing Agricultural University, No.1 Weigang, Nanjing, 210095 Jiangsu China; 3 Laboratory of Horticultural Science, Nagoya University, Nagoya, 464-8601 Japan

**Keywords:** Plant molecular biology, Plant physiology

## Abstract

Pear [*Pyrus bretschneideri* cv. Dangshan Su] fruit quality is not always satisfactory owing to the presence of stone cells, and lignin is the main component of stone cells in pear fruits. Caffeoyl shikimate esterase (CSE) is a key enzyme in the lignin biosynthesis. Although *CSE*-like genes have been isolated from a variety of plant species, their orthologs are not characterized in pear. In this study, the *CSE* gene family (*PbCSE*) from *P. bretschneideri* was identified. According to the physiological data and quantitative RT-PCR (qRT-PCR), *PbCSE1* was associated with lignin deposition and stone cell formation. The overexpression of *PbCSE1* increased the lignin content in pear fruits. Relative to wild-type (WT) *Arabidopsis*, the overexpression of *PbCSE1* delayed growth, increased the lignin deposition and lignin content in stems. Simultaneously, the expression of lignin biosynthetic genes were also increased in pear fruits and *Arabidopsis*. These results demonstrated that *PbCSE1* plays an important role in cell lignification and will provide a potential molecular strategy to improve the quality of pear fruits.

## Introduction

Pear (*Pyrus*) is one of the most important fruits commodities around the world, especially in China, and its cultivated area and yield accounts for 69% and 68% of the total global value, respectively (FAO, 2017). However, the quality of pears is not always satisfactory due to genetic variability and poor growing conditions, therefore the export price of Chinese pears is lower than the market average. The stone cells is an important factor affecting the quality of pears, especially ‘Dangshan Su’ pear, a *Pyrus* specie originating from China, has a lot of stone cells^1^. Stone cells are specialized cells in pears, which are lignified from parenchyma cells^2^. As one of the main components of stone cells, lignin affects the formation of stone cells in pear fruits^1,3^. Therefore, for great economic significance, the formation mechanism of lignin has been a focus of research.

Over the past few decades, the mechanism of lignin biosynthesis has been gradually become clear. Many enzymes have been identified to be involved in the biosynthesis of lignin monomers, such as cinnamate 4-hydroxylase (C4H), hydroxycinnamoyl-coenzyme A shikimate/quinate hydroxycinnamoyl (HCT), peroxidase (POD) and laccase (LAC)^4,5^. HCT plays a significant role in the lignin biosynthetic pathway, it can convert p-coumaroyl-CoA into p-coumaroyl shikimate. C3H convert p-coumaroyl shikimate into caffeoyl shikimate, and then HCT converted caffeoyl shikimate into caffeoyl-CoA. CSE, another important enzyme which recently discovered in lignin biosynthesis, can convert caffeoyl shikimate into caffeoyl-CoA through 4-coumarate: Coenzyme A ligase (4CL) without performing a second HCT reaction^6^. *CSE* was firstly identified from *Arabidopsis thaliana* and found that it can selectively hydrolyze caffeoyl shikimate into caffeate^6^. Recent studies have found that CSE is important for the lignification process in *Arabidopsis*, hybrid poplar (*Populus tremula* × *Populus alba*) and *Medicago truncatula*. A lack of *CSE* in *Arabidopsis*, hybrid poplar and *M. truncatula* result in a decrease in the lignin content^6–8^. Liu et al.^9^ confirmed that CSE has a role in endocarp lignification in peach (*Prunus persica L.*) fruits. However, there is no ortholog of the currently characterized *CSE* genes identified in the model grass *Brachypodium distachyon*, corn (*Zea mays)* and rice (*Oryza minuta*). Moreover, crude protein extracts of these species exhibit only a weak esterase activity with caffeoyl shikimate^7,10^, and it is hard to identify their functions. Overall, although the function of *CSE* has been confirmed in some plants, the general function of this enzyme is not clear because of the apparent lack of CSE activity in the lignified tissues of other plant species. The purpose of this study was to investigate whether *CSE* plays a role in cell lignification in pear fruits.

All members of the *CSE* family (*PbCSEs*) in pear may have different roles during plant growth and the genes related to cell lignification in pear fruits need to be identified. The purpose of this study was to identify the members of the *PbCSE* family in pear and determine the genes involved in the cell lignification of pear fruits. The results of this study will validate the role of the candidate genes and lay the foundation for cultivating pear fruits with fewer stone cells and better tasty varieties.

## Results

### Bioinformatics analysis of *PbCSE* family members

**Figure 1:**
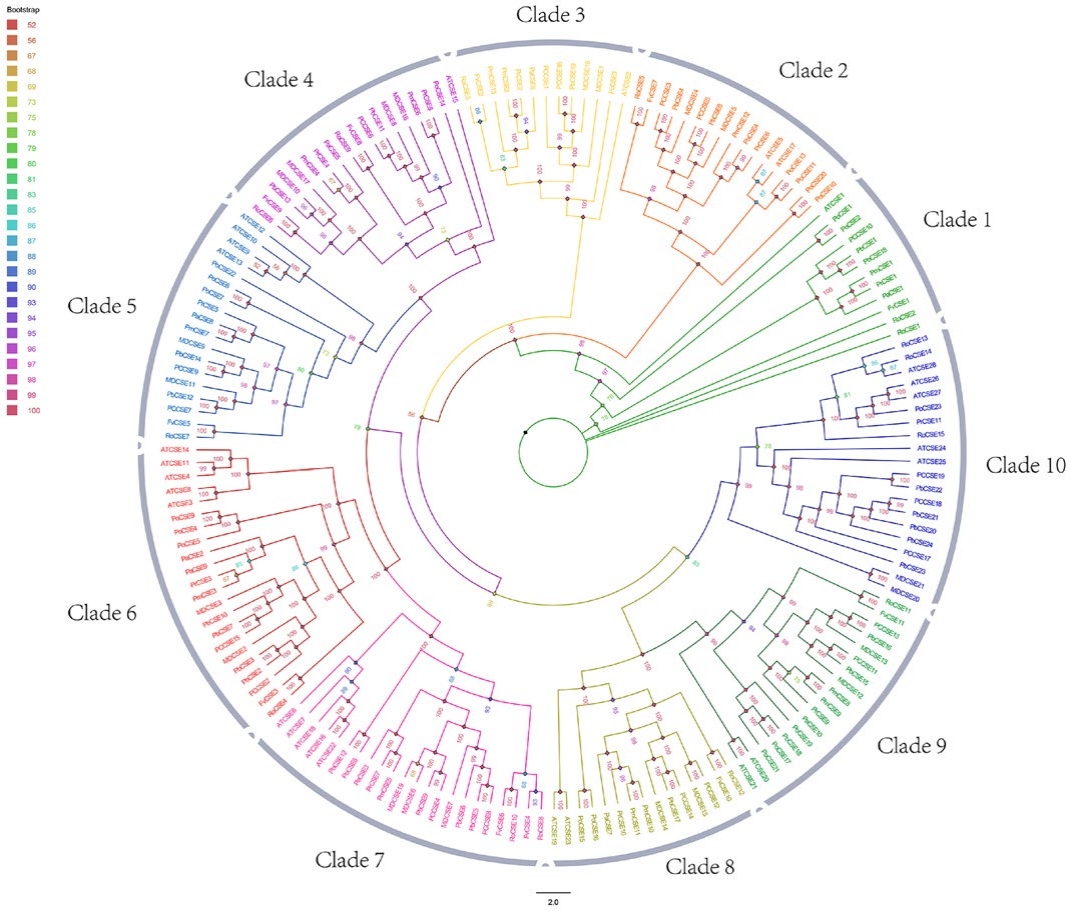
Phylogenetic analysis of CSE proteins from different plant species. The ten plant species included *Arabidopsis thaliana*, *Pyrus communis*, *Pyrus bretschneideri*, *Fragaria vesca*, *Malus*, *Mume*, *Cerasus*, *Amygdalus*, *PopulusL* and *Rubus*. The protein sequences were aligned and a phylogenetic tree was constructed using the maximum likelihood (ML) method and IQ-TREE1.6.9 software. The number (42–100) indicated the confidence level, which varies by its color. The information about the *CSE* genes in ten species from the family evolution tree was listed in Supplementary Table S3 online.

The *CSE* Hidden Markov Model (HMM) configuration file (PF12146) were used to identify each species specific Hydrolase_4 domain. After the Python script extracts the amino acids sequences from protein fasta file, new HMM configuration files were hmmbuilt for each species. The hmmscan with Pfam-A database and online site SMART (http://smart.embl-heidelberg.de/) were used to analyze protein sequences of candidate genes and to determinethe presence of the *CSE* domain. After BLASTP comparing with *AtCSE* (*AT1G52760.1*) to determine the similarity, 24 putative *CSE* genes in pear were identified. The basic information about the 24 *PbCSE* genes can be found as Supplementary Table S1 online. To investigate the relationships between the CSE family members, the phylogenetic tree including ten species was constructed, a total of 177 *CSE* proteins (Fig. 1). All the *CSE* family genes can be divided into ten evolutionary branches according to the topology of the phylogenetic tree, Gene structure and conserved motifs.

**Figure 2:**
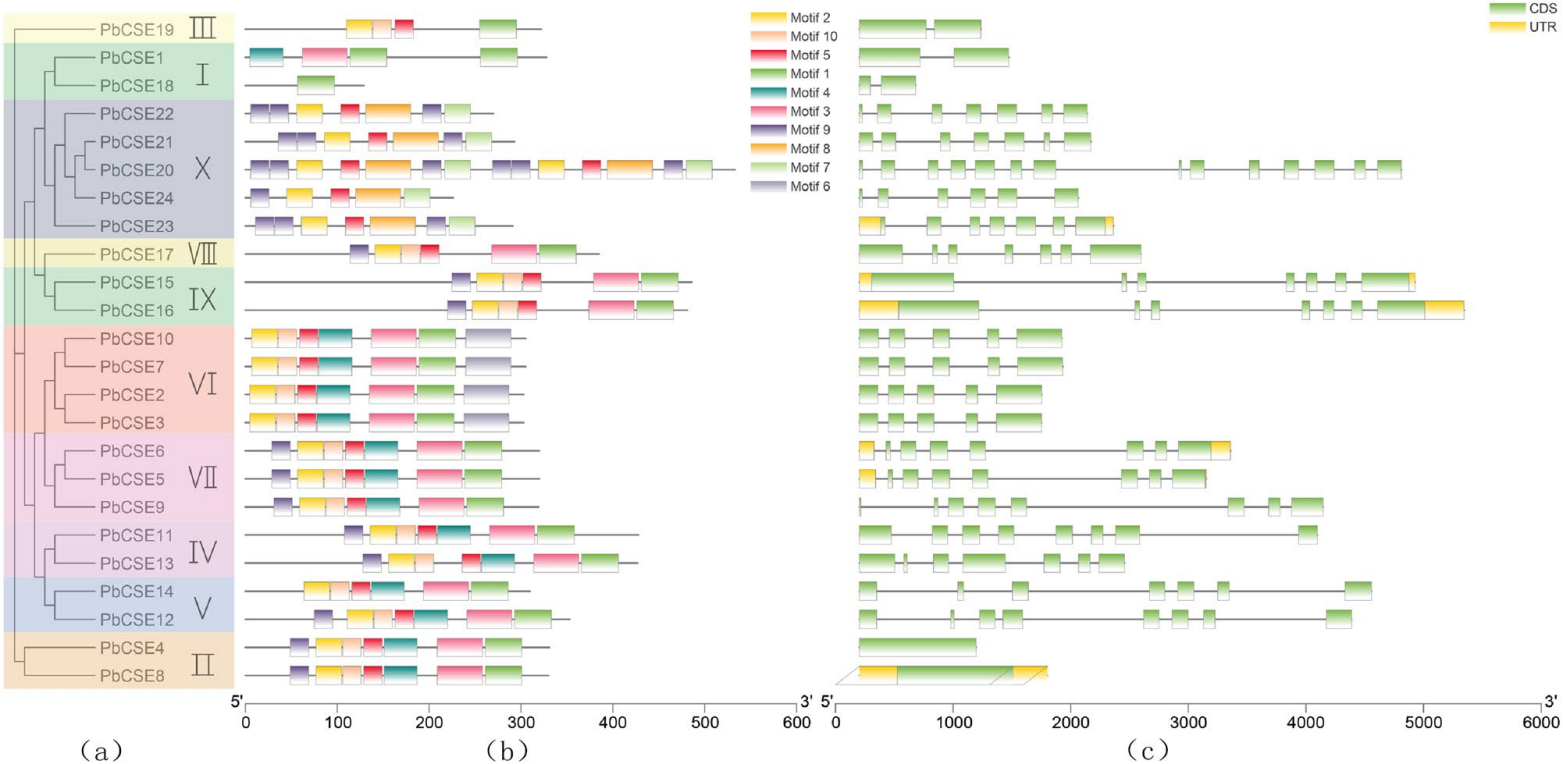
Gene characteristic analysis and chromosome localization of *PbCSE* proteins. (**a**) Phylogenetic analysis of *PbCSE* proteins. (**b**) The conserved motifs of *PbCSEs* based on their evolutionary relationships. (**c**) The gene structures of *PbCSEs* based on their evolutionary relationships.

Interestingly, both the type and distribution of the N-terminal and C-terminal domains were observed to have some family specificity. For example, motif 6 was present in the members of clade 6. The members of clade 1, 3 and 6 did not contain motif 9. In addition, the N-terminal and C-terminal domains of the members of clade 2, 4, 7–10 were identical in type and distribution. The N-terminal domain of the members of clade 6 lack motif 9 when compared to the members of clade 7 (Fig. 2a,b). An exon–intron structure evolution map was obtained using the GSDS server. The number of introns varies from 0 to 13, *PbCSE4* and *PbCSE8* have no introns and *PbCSE20* contained the most introns (Fig. 2c).

### The expression pattern of *PbCSE1* was consistent with the changes of stone cells and lignin contents

**Figure 3:**
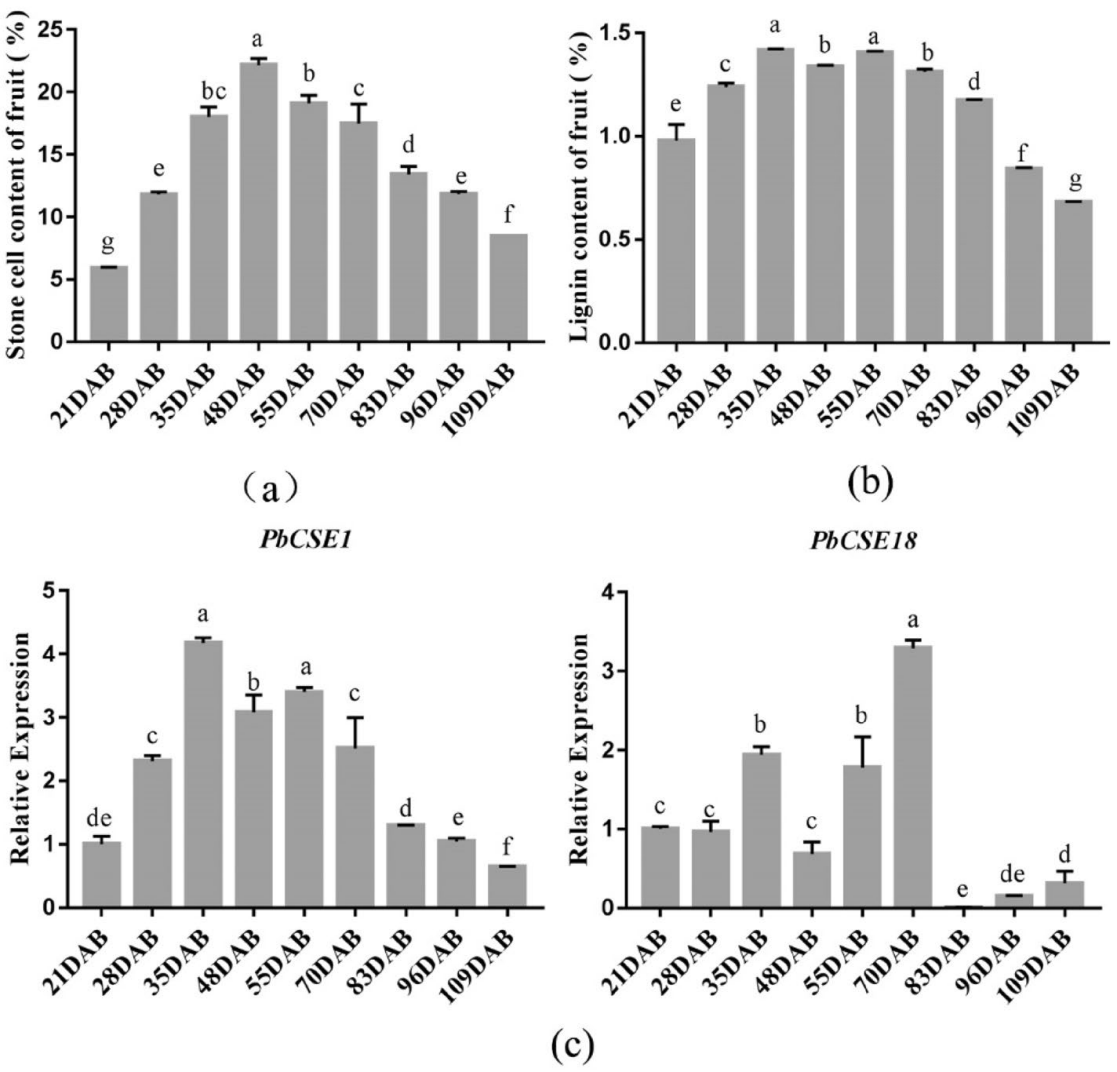
Expression analysis of the *CSE*-like genes during the formation of pear fruit lignin. (**a**) Stone cell content in ‘Dangshan Su’ pear during the growth phase. (**b**) Lignin content in ‘Dangshan Su’ pear during the growth phase. (**c**) A comparison of the relative expression levels of *PbCSE1* and *PbCSE18* during lignin synthesis. Different lowercase letters on a column indicated the treatment differs significantly at the 1% level.

The formation of stone cells and lignin deposition mainly occurs in the early stages of pear fruit development^11^. In this study, during 21 to 48 DAB, the stone cell content in the fruit increased rapidly and reached its peak at 48 DAB (Fig. 3a). The lignin content in the stone cells of pear fruit was abundantly expressed at 35 DAB and 55 DAB (Fig. 3b). According to previous studies, stone cells in pear reach a peak at ~ 48 DAB, while the lignin content was highest at ~ 35 DAB^11,12^.

Here, *PbCSE1* (*Pbr002315.3*) and *PbCSE18* (*Pbr002316.1*) had higher homology with *AtCSE1* (*At1g52760.1*). To validate the expression profiles of the two genes in pear fruits, we tested the relative expression levels of the two *PbCSEs* in pear using qRT-PCR. Interestingly, the relative expression of *PbCSE1* in fruit was consistent with the relative expression levels of lignin in pear, but *PbCSE18* was obviously inconsistent (Fig. 3c).

### Transient expression of *PbCSE1* changed the lignin content in pear fruits

**Figure 4:**
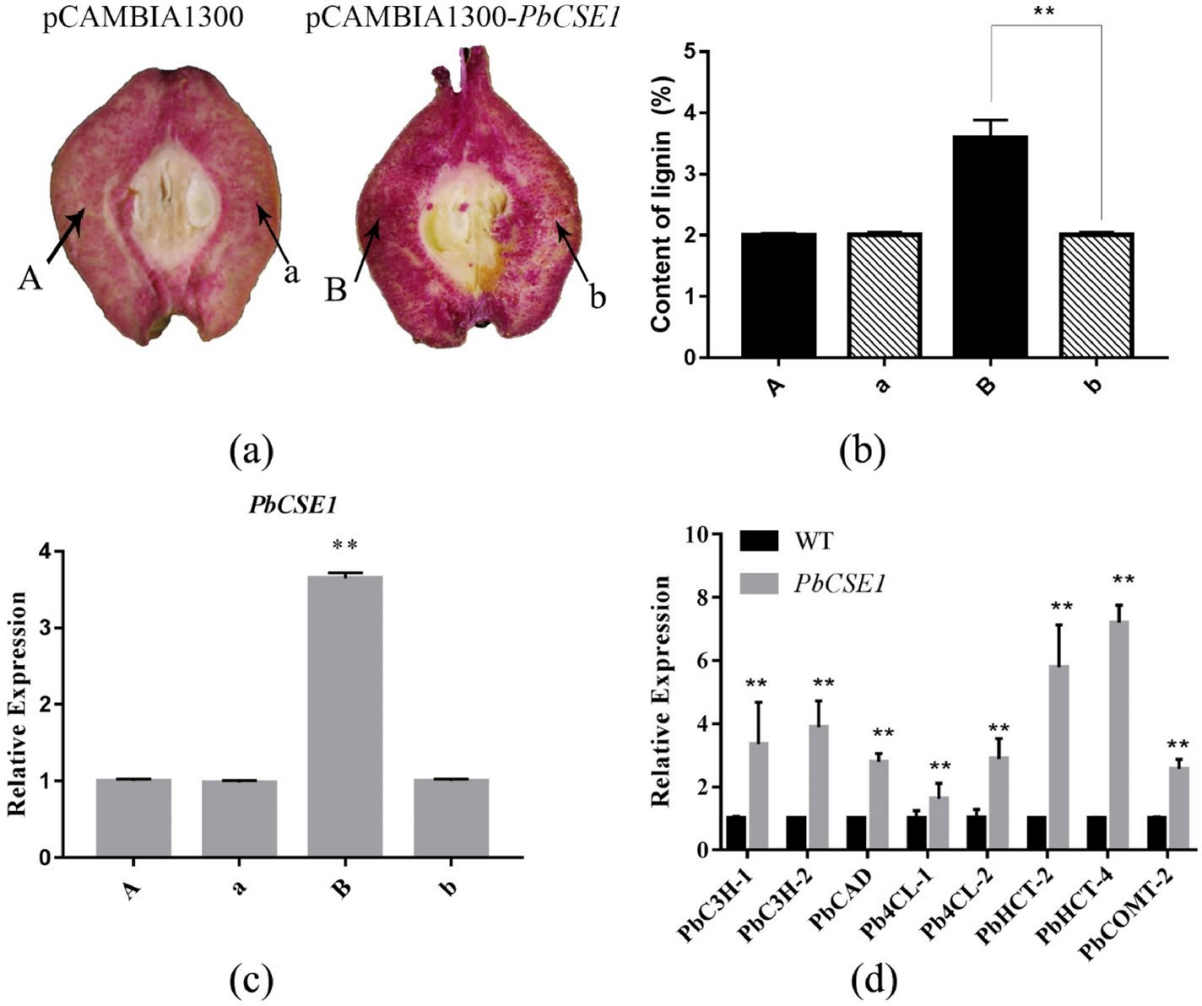
Expression analysis of the *CSE*-like genes in transgenic pear fruits. (**a**) Transient assays using *PbCSEs* overexpression in ‘Dangshan Su’ pear at 35 DAB. The infiltration sites were labelled A and B for the different gene constructs and its non-infiltrated sites were labelled a and b. The image was taken 10 d after agro-infiltration. (**b**) The lignin content in the fleshy tissue around the infiltration sites (A and B) and its non-infiltrated sites (**a**,**b**). (**c**) The expression levels of *PbCSE1* in the fleshy tissue around the infiltration sites and their respective non-infiltrated sites using q-PCR analysis. The asterisks indicate values that were determined by the t-test to be significantly different from their equivalent control (**P < 0.01). (**d**) The expression level of lignin synthesis-related genes in the overexpression of *PbCSE1* in pear fruits. The genes selected for expression analysis have been previously shown to be highly expressed in the stems where lignified fibers and vessels are abundant^13^. The asterisks indicate values that were determined by the t-test to be significantly different from their equivalent control (**P < 0.01).

To further elucidate the role of *PbCSE1* in cell lignification, the inoculum was infiltrated into pear fruits (35 DAB). After 10 d of infiltration, a strong increase in the lignin staining was observed at the injected points (B) of *PbCSE1* when compared with corresponding non-injected points (b) (Fig. 4a). It was apparent that the lignin content in the fleshy tissue around the injected points (B) of *PbCSE1* increased by 26.9% (**P < 0.01) when compared with the corresponding non-injected points (b), but no increase was evident in plants injected with the control vector (A and a) (Fig. 4b). The expression of *PbCSE1* at the injection site was analyzed using qRT-PCR. A significant up-regulation of *PbCSE1* expression in the corresponding transiently overexpressed pear fruit was observed (Fig. 4c).

*PbC3H1*, *PbC3H2*, *PbCAD*, *Pb4CL1*, *Pb4CL2*, *PbHCT2*, *PbHCT4* and *PbCOMT2* are related to lignin biosynthesis^13^. To elucidate the molecular mechanism of *PbCSE1* in pear fruits, we used qRT-PCR to analyze these genes in the overexpression of pear fruits. Compared to the control group, the expression level of lignin biosynthetic genes was significantly increased, while *HCT* and *C3H* were highest expressed in pear fruits (Fig. 4d).

### Overexpression of *PbCSE1* increased lignin deposition in transgenic *Arabidopsis* plants

In the T1 generation, five hygromycin-resistant transgenic plants were identified (0E6, OE8, OE11, OE21 and OE24; see Supplementary Fig. S1a online). The qRT-PCR results showed that when compared to the WT, the relative expression of *PbCSE1* was significantly increased and the relative expression levels of OE6, OE8 and OE11 exhibited higher levels of *PbCSE1* expression (see Supplementary Fig. S1b online). The T2 plants seed germination rate was close to 100%, which were taken from T1 transgenic plants.

**Figure 5:**
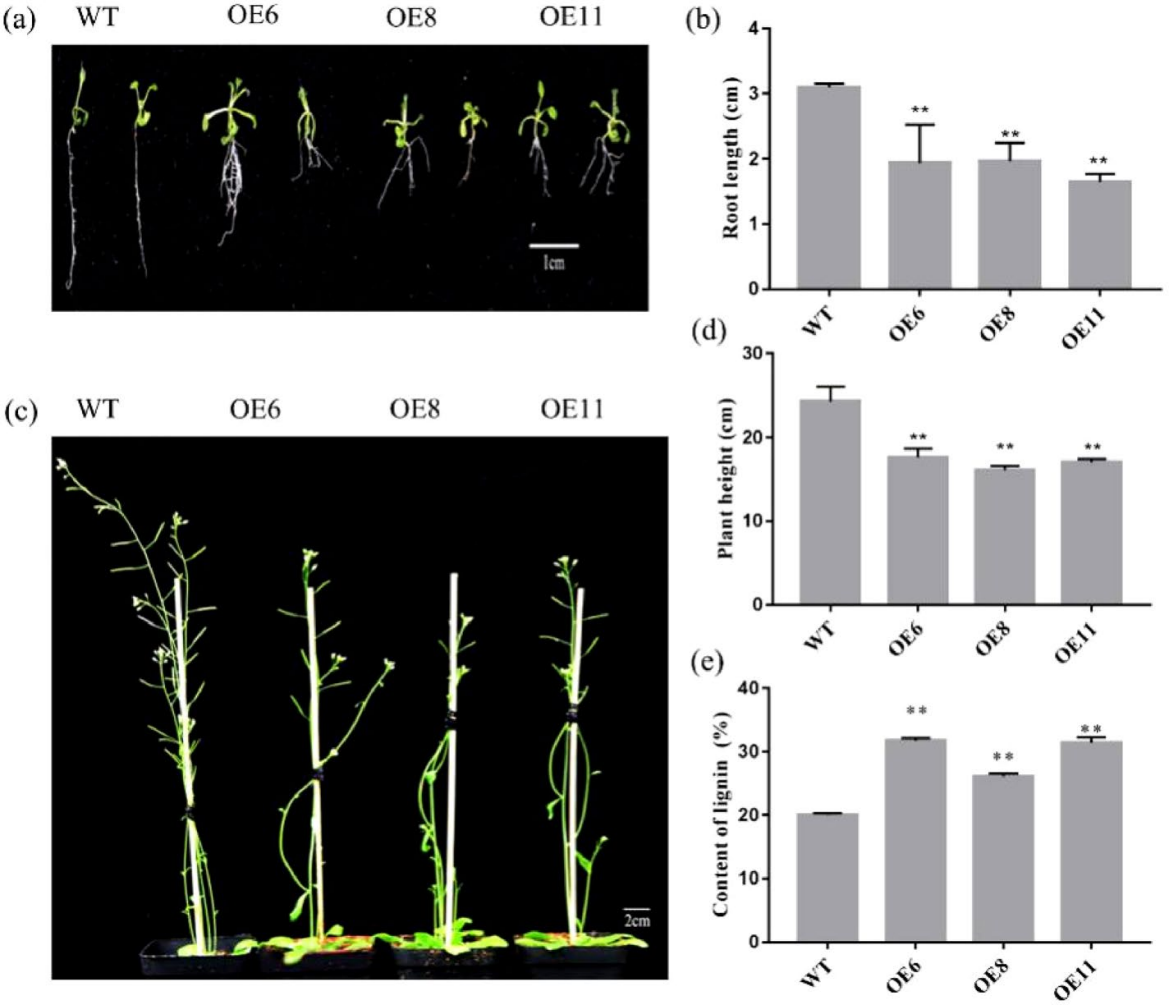
Identification of transgenic *Arabidopsis* positive seedlings and determination of the biomass from WT and *PbCSE1* transgenic *Arabidopsis* plants. (**a**) The root length of *PbCSE1* after 7 d. (**b**) The statistics of root length after 7 d. (**c**) Plant height of *PbCSE1* after 5 weeks. (**d**) The statistics of plant height after 5 weeks. The asterisks indicated values that were determined by the t-test to be significantly different from their equivalent control (**P < 0.01). (**e**) The lignin content in the WT and overexpressing *Arabidopsis* lines.

**Figure 6:**
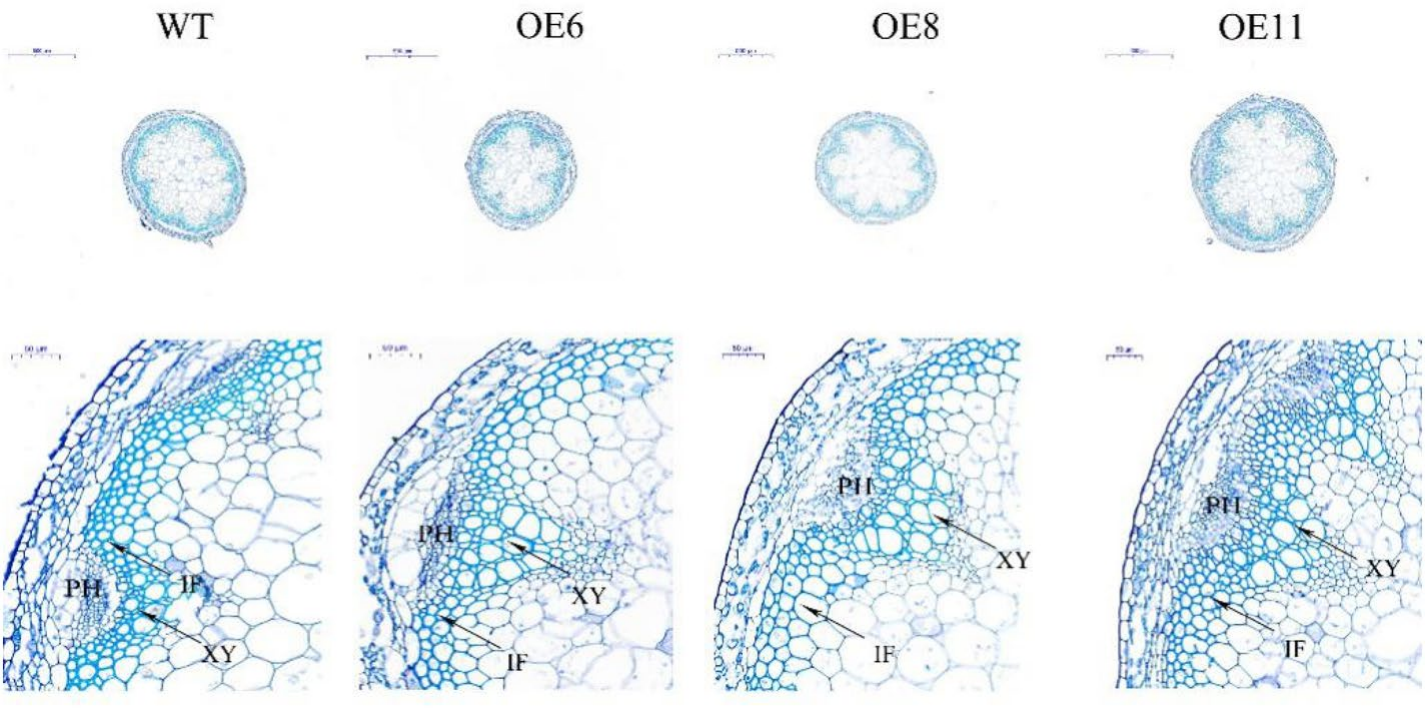
Sections stained with toluidine blue, showing the variation of cell walls in *Arabidopsis* with *PbCSE1* overexpression. *PH* phloem, *XY* xylem, *IF* interfascicular fiber.

WT and transgenic T3 seeds were planted in MS medium under the same growth conditions and the root lengths of 50 seedlings were evaluated one week later. The development of the root was significantly suppressed, as shown in Fig. 5a. The root length of the *PbCSE1* transgenic line decreased by an average of 1.219 cm (**P < 0.01) when compared to the WT (Fig. 5b). The transgenic lines and WT were then grown under long daylight conditions. During the flowering stage (5 weeks), the transgenic lines were later flowering than the WT, and the inflorescence stems became thick and short (Fig. 5c). The stem length of the *PbCSE1* transgenic line was decreased by an average of 6.923 cm (**P < 0.01) when compared to the WT (Fig. 5d). The lignin content of the stems in the transgenic *Arabidopsis* lines were significantly higher than that in the WT, especially OE6 was the highest (31.74%), which was 11.75% higher than those of the WT (Fig. 5e). In order to examine whether the expression of *PbCSE1* in *Arabidopsis* leads to specific lignification in stems, the sections of stems were stained with toluidine blue to observe the lignin of WT and transgenic plants. There were some changes in the morphology and amount of vessel cells in the transgenic lines, the xylem tissue of the transgenic lines showed a stronger lignification than that of the WT (Fig. 6). These results indicated that *PbCSE1* promoted lignin deposition.

**Figure 7:**
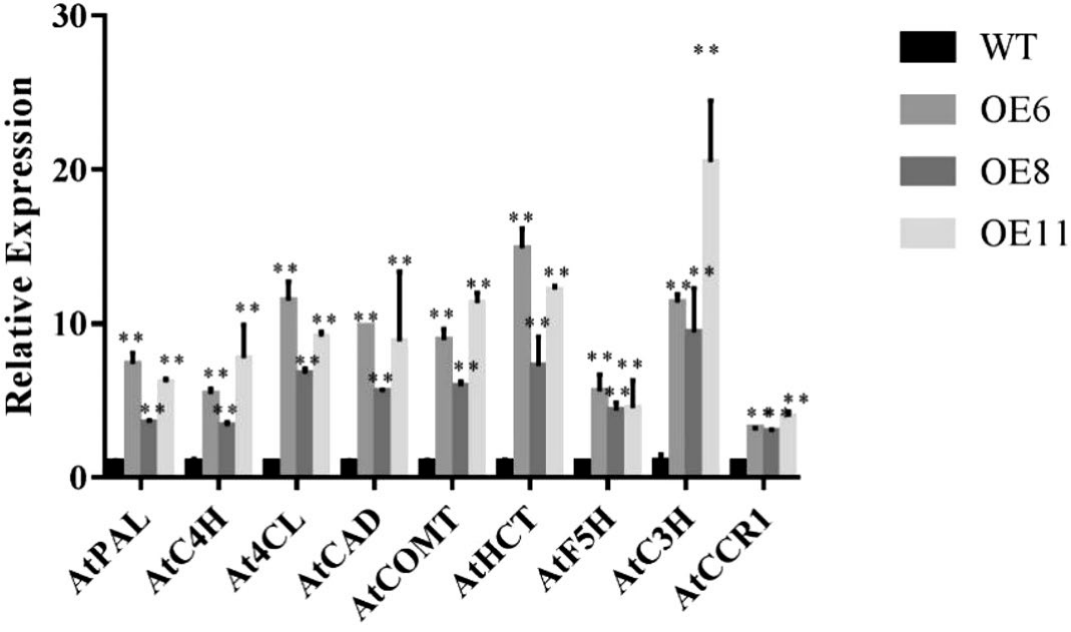
The expression of lignin biosynthesis-related genes in *Arabidopsis* along with *PbCSE1* overexpression (** *p* < 0.01). The genes selected for expression analysis have been previously shown to be highly expressed in stems where lignified fibers and vessels are abundant^14^.

The A*rabidopsis* loss-of-function *CSE-2* mutant down-regulated all upstream genes *(AtPAL, AtC4H, AtC3H, At4CL, AtCCR, AtCOMT, AtF5H, AtHCT* and *AtCAD)*^14^. To elucidate the molecular mechanism of *PbCSE1* during lignin synthesis, we used qRT-PCR to analyze lignin biosynthetic genes in the overexpression of stems in *Arabidopsis*. When compared to the WT, the expression level of lignin biosynthetic genes was significantly increased, while *HCT* and *C3H* were highest expressed in the stems of transgenic *Arabidopsis* (Fig. 7).

## Discussion

Stone cells are an important factor determine the quality and value of pear fruits worldwide^3^. The formation of stone cells is highly correlated with lignin biosynthesis and deposition in pear fresh^15^. CSE is an enzyme that catalyses lignin monomer synthesis, it can form an alternative pathway to caffeyl-CoA with 4CL. A lack of *CSE* in some plants results in a decrease in the lignin content as well as its structure^6–8^. Recent studies have focused on the verification and regulation of lignin synthesis structural genes based on the pear genome and germplasm resources^4,16^, the mechanism of lignin synthesis need to be further studied.

Some *CSE* orthologs have been found primarily in model plants, but the function and underlying mechanisms of the *CSE* genes are elusive in perennial fruit trees. In our study, 24 *CSEs* orthologs were found in pear genome. In the phylogenetic tree, the number of *PbCSEs* were fewer than *Arabidopsis* (28), and more than *Pyrus communis* (19), *Fragaria ananassa* (11), *Malus* (21), *Armeniaca mume* (13), *Cerasus* (10), *Amygdalus* (11), *Populus* (23) and *Rubus* (15). These results indicated that different replication events and expansion mechanisms had occurred in the *CSE* family of different species. Protein motif analysis suggested that the differences in the composition of the N-terminal domain may confer different biological functions to each of the members in the evolutionary branch. Gene structure and collinear analyses of *PbCSEs* showed family specificity. These results showed that most of the *PbCSE* members in the same clade had similar functions.

An orthologous is a homologous gene that differentiates after a speciation event. It is generally assumed that orthologous genes retain the same function in different organism because homology pairs (groups) are produced by evolution, and then ancestral genes and their functions are maintained by speciation events. CSE is important for the lignification process in *Arabidopsis*^6^. The *CSE* family phylogenetic tree showed that *AtCSE1*, *PbCSE1* and *PbCSE18* belonged to the same evolutionary branch, indicating that they may have similar biological functions. *AtCSE1* had an important role in the lignification process^6^. Ha et al.^7^ have shown that *MetCSE (Medtr4g127220)* plays an essential role for lignification in *M. truncatula.* There is a research have been reported that *PbCSE1* has a closer evolutionary relationship to *AtCSE1*, *MetCSE* and may be involved in lignin biosynthesis of pear fruit^17^. In this study, the relative expression of *PbCSE1* was consistent with the relative expression of lignin in pear, therefore, we assumed that *PbCSE1* was a candidate gene that plays an important role in the lignification of pear fruits. *Arabidopsis* is a model plant with a stable expression system, a variety of plant genes have been heterologously expressed in *Arabidopsis* to verify its function^18,19^. In this study, compared to the WT, transgenic *PbCSE1 Arabidopsis* inhibited plant growth and development; promoted lignification of xylem tissue; and the lignin contents of the stems were increased significantly. These results demonstrated that *PbCSE1* promotes lignification during stone cell development in pear fruit, consistent with the reported functional results of *AtCSE1*. Lignin deposition frequently has a negative effect on plant growth and development^20^. It may because of these modifications to the lignin deposition or conversely as a consequence of the accumulation of certain pathway intermediates detrimental to growth or by-products^21^. The method of fruit transient expression has been successfully verified in fruits such as banana, strawberry and tomato^22–24^. In this study, we used the same method of transient expression in pear fruit. When compared with the control group, the staining of the lignin was significantly increased and the lignin content increased by 26.9% (**P < 0.01) after transient overexpression of *PbCSE1*. It was apparent that transient expression of *PbCSE1* changed the lignin content in pear fresh.

Many studies have expressed lignin synthesis key genes in transgenic plants and observed their expression trends^18,25^. In *Arabidopsis*, loss-of-function *CSE-2* mutant down-regulated lignin biosynthetic genes^14^. These suggest that the reason of changes in lignin contents may be the combination of these genes promotes the biosynthetic pathway or the mono-lignin precursor increases the expression levels of these genes. In our study, the expression levels of lignin biosynthetic genes were increased in transgenic pear fruits and the stems of *Arabidopsis*. These genes may work synergistically to promote lignin synthesis, of which *HCT* and *C3H* may play key roles. Second-level MYB master switches (*MYB-46* and *MYB-83*) and lignin-specific activators (*MYB-63* and *MYB-85*) can effectively activate the *CSE* promoter in *Arabidopsis*^14^, but there is no related report in pear until now and the mechanism of their interaction needs to be further studied.

Taken together, these results suggested that *PbCSE1* promoted cell lignification, increased the expression levels of lignin biosynthetic genes and the lignin contents, and clarified that *PbCSE1* may play a positive role in lignin biosynthesis in pear. This study will help to further reveal the regularity of pear fruit cell formation and lay the foundation for further studies on the regulation mechanism of pear fruits.

## Materials and methods

### Plant materials

In this study, *Pyrus bretschneideri* cv. ‘Dangshan Su’ which were planted at Nanjing Agricultural University Experimental Orchard (Baoying City, Jiangsu Province, China) under uniform management, were selected as the plant materials. Samples of the pear fruits were collected at 21, 28, 35, 48, 55, 70, 83, 96 and 109 days after blossom (DAB) from five individual trees. After being brought to the laboratory, the pear fruits were peeled and cut into small pieces and blended. The blended flesh was then divided into two batches, one was used for the determination of physiological indicators. The other was packaged, frozen using liquid nitrogen and then stored in a refrigerator at − 80 °C for subsequent tests.

### Plant material statement

We declare that the plant material in the experiment was collected and studied in accordance with relevant institutional, national, and international guidelines and legislation. All the experimental materials were stored in the Life Science Building of Nanjing Agricultural University.

### Sequence search and annotation of *CSE* genes

To identify CSE genes from pear and the nine other species, arabidopsis (*Arabidopsis thaliana*), peach (*Prunus persica*), apple (*Malus domestica*), strawberry (*Fragaria vesca*), black raspberry (*Rubus occidentalis*), sweet cherry (*Prunus avium*), aspen (*Populus trichocarpa*), mumeplant (*Prunus mume*) and European pear (*Pyrus communis*), several approches were employed. The sequences of the *CSE* family in 10 species were downloaded from previously reported databases (https://www.Arabidopsis.org; https://www.rosaceae.org; https://phytozome.jgi.doe.gov)^3^. Using a *CSE* family characteristic domain (Pfam: PF12146) as a query model in accordance with the HMM configuration file, we anchor for each species domain using the HMM with E-values < 1e−10 and HmmBuild new characteristic domain model for each species. Then, the candidate genes were searched for each species by HMM with new models. The HMM profile (PF12146) was downloaded from www.pfam.com. HmmScan was used to determine the domain PF12146 in candidate genes with Pfam-A database and the candidate were BLASTP with *AtCSE* (*AT1G52760.1*) to determine the similarity, which E-values < 1^11,26^.

### Phylogenetic analysis and protein analysis.

The InterProScan program was used on all the candidate protein pairs and confirmed the presence of the diagnostic domain using the Pfam and SMART databases. DNAMAN used default parameters to align multiple homologous *CSE* genes (https://blast.ncbi.nlm.nih.gov/Blast.cgi). A phylogenetic tree was constructed using the maximum likelihood method and IQ-TREE 1.6.9 software. The guide values displayed next to each branch were inferred from 1000 replicate trees. The open reading frame (ORF) program of the gene structure display server (GSDS) (http://gsds.cbi.pku.edu.cn/) was used with the genomic sequence (http://gsds.cbi.pku.edu.cn/)^27^.

### Chromosomal location, gene structure of the *PbCSE* family

The chromosomal locations of *PbCSE* genes were based on genomic annotated data. We then used CIRCOS to produce a circular visualization of the *CSE* genes, which were mapped onto the different chromosomes^28^ (see Supplementary Fig. S2 online). To gain detailed information on the protein motifs, we used MEME to identify the conserved motifs, which were shared among the *PbCSE* proteins^29^. The parameters were: maximum number of different motifs = 10, minimum motif width = 6 and maximum motif width = 50.

### Stone cell content in pear flesh

The stone cell content was measured using a freeze-HCl method^1^. The fruits at different periods were peeled and the flesh reserved for testing. 100 g of flesh obtained from in each period was frozen at − 20 °C for 24 h, then thawed and homogenized with distilled water in a stirrer for 10 min. The sample was stirred for 1 min, allowed to stand for 3 min and then the upper layer of the suspension was discarded. The remaining suspension was precipitated for 30 min using 0.5 mM HCl, decanted and washed with distilled water. The precipitate was dried to constant weight and weighed. The stone cell content was calculated as follows: Stone cell content (%) = weight of stone cells (g DW) / weight of flesh (g FW) × 100%.

### Lignin determination

An acetyl bromide method was used to detect lignin in pear^1^. Pear fruit flesh obtained at different periods, *Arabidopsis* stalks and the transient expression of flesh were dried in the ovenand ground into a powder. Among them, the transient expression of flesh was divided into infiltratedparts and non-infiltrated parts for processing. 0.01 g of the samples (3 replicates per sample) were prepared to determine the lignin content. The samples were ground with 95% ethanol, washed 3 times with 95% ethanol and ethanol:hexane (1: 2, v / v), and dried. The dried pellet was digested in 2 mL of a 25% (v/v) acetyl bromoacetic acid solution and reacted at 70 °C for 30 min. The reaction was stopped by adding 0.9 mL of 2 N NaOH followed by 5 mL of acetic acid and 0.1 mL of 7.5 M hydroxylamine hydrochloride. The volume was made up to 10 mL with acetic acid and the absorbance at A280 was determined. Finally, the lignin content was determined using a lignin standard sample curve^1^. The lignin content was reported as a percentage (calculated lignin content/dry weight of stone cells × 100%)^30^. Three independent experiments were performed (at least ten fruits were used in each experiment).

### RNA extraction and qRT-PCR analysis

RNA was extracted from the samples using a polysaccharide polyphenol plant RNA extraction kit (FUJI, Chengdu, China). Using a NeuScript II 1st strand cDNA synthesis kit (NUOWEIZAN, Nanjing, China) to reverse transcription RNA. The RNA and DNA samples were quantified using a Nanodrop spectrophotometer (THERMO, Massachusetts, USA). The primer sequences of quantitative real-time PCR (qRT-PCR) were shown in Supplementary Table S2 online. To observe the trend in the gene expression, the genes were identified using qRT-PCR analysis. About 1 μg of total RNA was used as a reverse transcription template using a ReverTra Ace-α 1st strand cDNA synthesis kit (TOYOBO, Osaka, Japan) according to the manufacturer's instructions. All reactions were repeated three times in 96-well plates on a Roche LightCycler 480II quantitative PCR machine. The relative expression levels were calculated using the 2^-ΔΔCt^ method. Roche LightCycler 480 SYBR GREEN I Master fluorescent dye was used for qRT-PCR (TAKARA, Dalian, China). Tubulin was used as an internal reference gene for pear and AtActin was used for *Arabidopsis*.

### Gene cloning and vector construction

CE Design V1.03 software was used to design the primer pairs containing Xba I and BamH I restriction sites among the pCAMBIA1300 vector. The primer sequences of real-time PCR (RT-PCR) were shown in Supplementary Table S2 online. Using the Phanta Max High-Fidelity PCR Enzyme (VAZYME, Nanjing, China) to clone the target gene. The product was took into the pCAMBIA1300 vector to generate a fusion construct (p1300-*PbCSE1*-GFP) using a One-step Rapid Cloning Kit (VAZYME, China). The fusion constructs were transferred to *E. coli* strain DH5α using a freeze–thaw method. After sequence confirmation, the fusion constructs and control vector (pCAMBIA1300) were transferred to *Agrobacterium tumefaciens* strain GV3101 through the same freeze–thaw method^31^.

### Transient expression of pear fruits

For improvingtransformation, the *Agrobacterium* cells were grownin Luria–Bertani (LB) medium and cultured at 28 °C with shaking at 200 rpm for 12 h. The cells were then centrifuged and re-suspended in an infiltration buffer (10 mM MgCl_2_, 0.2 mM acetosyringone, 10 mM MES, pH 5.5) with an OD_600_ value for 0.8–1.2. 0.1 mM Acetosyringonewas added, followed by incubation at 21 °C for > 4 h. The inoculum was injected into the flesh of ‘Dangshan Su’ pears at 35 DAB by needleless syringes. Each construction was repeated three times and the transformed fruits were placed in the dark at 21 °C overnight and then transferred into a growth chamber (21 °C, 16 h light/8 h dark) for 10 d under low light conditions, stained and photographed^32,33^. RNAs were extracted from pear fresh obtained from the infiltrated parts and non-infiltrated parts, and the expression of genes involved in phenylpropanoid biosynthesis were quantitatively determined using qRT-PCR (the primers were listed in Supplementary Table S2 online).

### Transformation and characterization of transgenic *Arabidopsis* plants

To further determine whether *PbCSE1* promotes cell lignification, *Arabidopsis* plants (ecotype Columbia) were transformed using the floral dip method^34^. The T1 generation seeds were screened in a transplanted workbench using a medium containing 30 g/L sucrose, 0.75% agar, 20 mg/L hygromycin, 100 mg/L timentin and 100 mg/L carbenicillin. The DNA of the transgenic plants was extracted using cetyltrimethyl ammonium bromide (CTAB) and the transgenic lines identified using PCR^13^. T2 generation seeds and wild-type (WT) seed species, identified as pure lines, were screened in MS medium as T3 transgenic plants. The growth morphology of the plant were observed during the seedling stage and plant growth period, respectively^35^. After 5 weeks, RNAs were extracted from the stems of transgenic *Arabidopsis* plants, and the expression levels of genes involved in phenylpropanoid biosynthesis were quantitatively determined using qRT-PCR (the primers were listed in Supplementary Table S2 online).

### Statistical analysis

The data were analyzed using the ANOVA program of SPSS (IBM SPSS 22), reflecting significance analyses (* and ** indicated *P* < 0.05 and 0.01, respectively). Microsoft Excel 2013 was used for statistical analysis, the data were presented as the mean ± SE of at least three independent replicates obtained from one experiment.

### Approval of animal use

Animal use was approved by National Taiwan University College of Medicine and College of Public Health Institutional Animal Care and Use Committee (IACUC) with the IACUC Approval Number 20170556. And all experiments in this study were performed in accordance with relevant guidelines and regulations.

## Supplementary Information

Supplementary Information 1.

Supplementary Information 2.
