# Supplementary material for: PbCSE1 promotes lignification during stone cell development in pear (Pyrus bretschneideri) fruit
Source: Sci Rep. 2021 May 3;11:9450. doi: 10.1038/s41598-021-88825-0 (PMC8093294; doi:10.1038/s41598-021-88825-0)
Supplement: Supplementary file 1 — Supplementary Information 1. [file 41598_2021_88825_MOESM1_ESM.pdf]

PbCSE1 promotes lignification during stone cell development in pear (*Pyrus bretschneideri*) fruit

Jiahui Xu[1], Xingyu Tao[1], Zhihua Xie[1], Xin Gong[1], Kaijie Qi[1], Shaoling Zhang[1,2], Katsuhiro Shiratake[3], Shutian Tao[1,2*]

[1] Pear Engineering Research Centre, College of Horticulture, Nanjing Agricultural University, Nanjing, Jiangsu Province 210095, China

[2] State Key Laboratory of Crop Genetics and Germplasm Enhancement, Nanjing Agricultural University, Nanjing, Jiangsu Province 210095, China

[3] Laboratory of Horticultural Science, Nagoya University, Nagoya, Japan, 464-8601

[*] Correspondence: taost@njau.edu.cn; Tel: +86-025-84999961

Jiahui Xu: 1491469981@qq.com

Xingyu Tao: 2018104025@njau.edu.cn

Zhihua Xie: xzz811@126.com

Xin Gong: 2016104030@njau.edu.cn

Kaijie Qi: qikaijie@njau.edu.cn

Shaoling Zhang: slzhang@njau.edu.cn

Katsuhiro Shiratake: shira@agr.nagoya-u.ac.jp

*Corresponding author: Shutian Tao, Centre of Pear Engineering Technology Research,

College of Horticulture, Nanjing Agricultural University, No.1 Weigang, Nanjing, Jiangsu, The People's Republic of China, 210095, Phone: +86-25-8439996, Fax: +86-25-84399961, E-mail: taost@njau.edu.cn.

Supplementary data

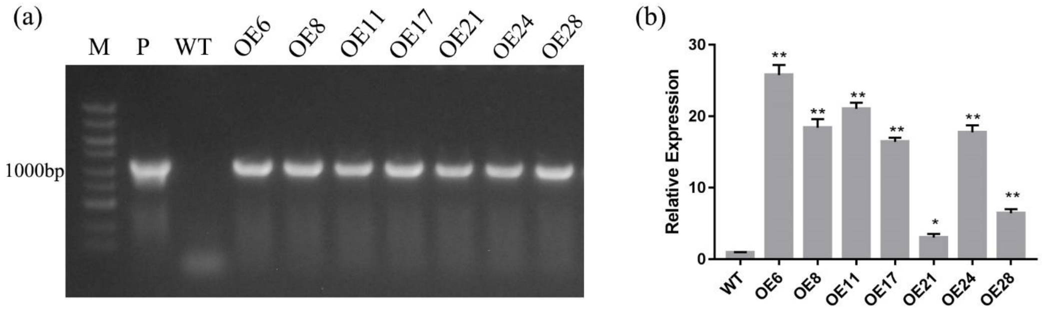

Figure S1. Identification of transgenic *Arabidopsis*-positive seedlings. (**a**) T1 generation positive seeding detection of *PbCSE1*. OE = overexpression, M = marker, WT = wild type, P = positive control. This is the grouping of gels cropped from different parts of the same gel. (**b**) The relative expression of *PbCSE1* in different lines of T1 positive plants.

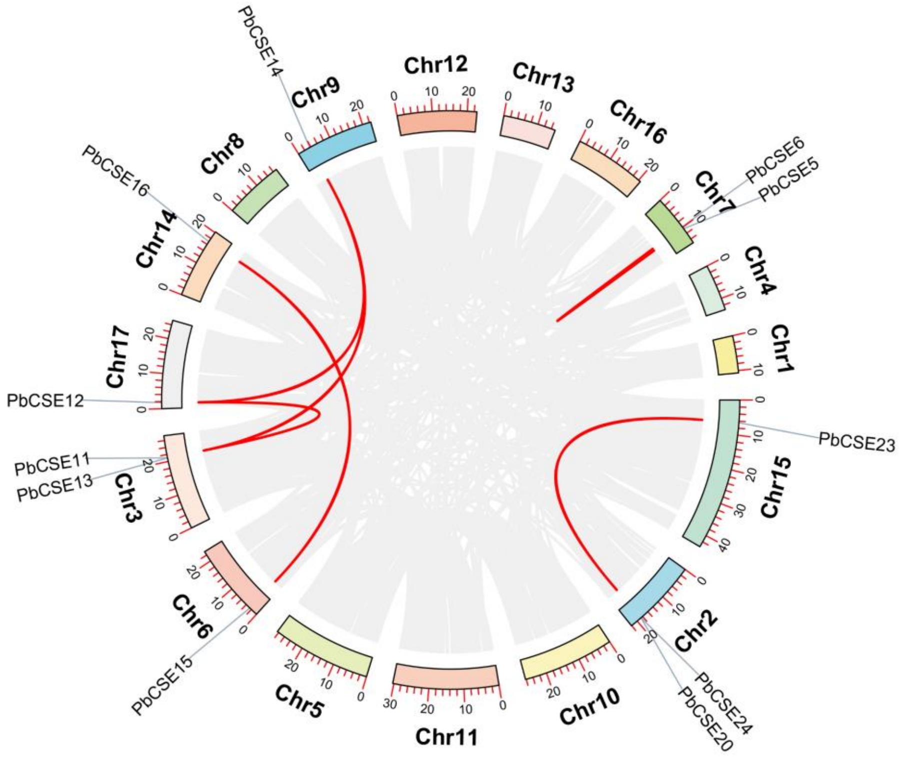

Figure S2. Collinear analysis of *PbCSEs*.

No.	Gene name	Gene ID	Position	Stra	Start	End	DNA/bp	CDS/bp
1	*PbCSE1*	*Pbr002315.3*	Chr17	+	417048	418317	1270	984
2	*PbCSE2*	*Pbr026316.1*	scaffold424.0	-	38552	40101	1550	909
3	*PbCSE3*	*Pbr026363.1*	scaffold424.0	+	335722	337270	1549	909
4	*PbCSE4*	*Pbr039057.1*	scaffold828.0	-	51217	52209	993	993
5	*PbCSE5*	*Pbr032308.1*	Chr7	+	10475405	10478354	960	957
6	*PbCSE6*	*Pbr026463.1*	Chr7	-	9780086	9783240	3155	960
7	*PbCSE7*	*Pbr003260.1*	Chr11	+	90243	91971	1729	915
8	*PbCSE8*	*Pbr042390.1*	Chr3	-	27244839	27246437	1599	990
9	*PbCSE9*	*Pbr038157.1*	scaffold797.0	+	161366	165308	3943	957
10	*PbCSE10*	*Pbr003259.1*	Chr11	+	78090	79811	1722	915
11	*PbCSE11*	*Pbr013306.1*	Chr3	-	21021920	21025814	3895	1284
12	*PbCSE12*	*Pbr034764.1*	Chr17	+	1718219	1722403	4185	1059
13	*PbCSE13*	*Pbr013307.2*	Chr3	+	21019023	21021277	2255	1281
14	*PbCSE14*	*Pbr032606.2*	Chr9	+	3640437	3644791	4355	930
15	*PbCSE15*	*Pbr028331.1*	Chr6	+	2620950	2625674	4725	1458
16	*PbCSE16*	*Pbr038811.1*	Chr14	+	16893331	16898472	5142	1443
17	*PbCSE17*	*Pbr008780.1*	Chr15	-	8768139	8770533	2395	1155
18	*PbCSE18*	*Pbr002316.1*	Chr17	+	418552	419030	479	387
19	*PbCSE19*	*Pbr000313.1*	Chr5	+	26316512	26317545	1034	966
20	*PbCSE20*	*Pbr041464.1*	Chr2	+	18579530	18584136	4607	1599
21	*PbCSE21*	*Pbr027746.2*	Chr2	-	16457823	16459791	1969	879
22	*PbCSE22*	*Pbr015620.1*	Chr15	+	14831830	14833767	1938	810
23	*PbCSE23*	*Pbr042754.1*	Chr15	+	6452652	6454811	2160	873
24	*PbCSE24*	*Pbr041469.2*	Chr2	+	18552536	18554396	1861	678

Table S1. Information of 24 *CSE* genes in pear.

Gene name	Primer sequences (5′-3′)	Application	Note
P1300-*PbCSE1F*	GAGAACACGGGGGACTCTAGAATGGTCATGTACTTCCAATCCC	cDNA cloning	Used in this study
P1300-*PbCSE1R*	GCCCTTGCTCACCATGGATCCTCAAATTCCCTCCTCCAAGCC		
PbCSE-1F	ATGGTCATGTACTTCCAATCCC	qRT-PCR	
PbCSE-1R	GGACTTTGCTGGGCTTCAT		
PbCSE-18F	ATGGACTACGTAGAATTCTTTG		
PbCSE-18R	CCCTATCCGTCGTCCCGTGT		
ATC4H-F	GGGAGAAATCAACGAGGACA		The genes selected for expression analysis have been previously shown to be highly expressed in stems where lignified fibers and vessels are abundant[14].
ATC4H-R	CACGTGCGATTCTTCTTCAA		
AT4CL-1F	GATGCCGTTAATGCCAAGTT		
AT4CL-1R	ACCGATGAGCAAAGCCTCTA		
ATC3H-1F	TTTCGTTGATGCGTTGCTAA		
ATC3H-1R	CGGTCAAGTCCAACCACTCT		
ATCOMT-F	TAGCCAGTGCTTCCGTTCTT		
ATCOMT-R	CTAGGGTCAGTCCCGTGGTA		
ATHCT-F	TTGTCATCCCCAGATTCCAT		
ATHCT-R	CCACAACGAAGAGAACACCA		
ATF5H-1F	ATGATGGGGATGTTGTCGAT		
ATF5H-1R	CGTCCATGATGATTGCTTTG		
ATCCR-1F	AGGGGCTCAAATCCTCTCTC		
ATCCR-1R	GCGATGGAATAAAGGCAAGA		
ATCAD-6F	GTCCACTGAGCCACTTTGGT		
ATCAD-6R	CCCAAGCATAAGCAGAGGAG		
ATPAL-1F	GGATTTTCCGGTATCCGATT		
ATPAL-1R	CACTCATCACCTCTGCGAAA		
PbCAD-F	CCTCCTTGTTGGTTGCTGC		The genes selected for expression analysis have been previously shown to be highly expressed in the stems where lignified fibers and vessels are abundant[13].
PbCAD-R	CCGCCTTGGGTGGTTTT		
PbCCOMT-2F	TACCCAAGGGAGCCTGAATC		
PbCCOMT-2R	AGTAGCCAGTGTAGACCCCAATC		
PbC3H-1F	TGATTACTGCGGGTATGGACA		
PbC3H-1R	AGCATTAGTGGTGTTGGAGGG		
PbC3H-2F	GAGTTCCGACCCGAAAGGT		
PbC3H-2R	ATAGGTGGCCCAACATAGAGG		
Pb4CL-1F	CTTGGACAGGGGTATGGAATG		
Pb4CL-1R	AGCGAAGCACCCGTATCAG		
Pb4CL-2F	CCTACCAATCTCATGCCGTCT		
Pb4CL-2R	GGTTCTGGAAGCAGTAGCTGTG		
PbHCT-2F	GACGCCAAAGAAGGCACTG		
PbHCT-2R	GGGTAGAACGGCACAAGAGC		
PbHCT-4F	GACCCACCTCAGACAACATT		
PbHCT-4R	CGTCAAGACGCTTAGTTGCTCCC		

Table S2. Sequences of the gene-specific primers used in this work.

Code	name	evalue with AT1G52760.1	Chr	head	end	chain
Ro06_G16613	*RoCSE1*	0	Ro06	11766242	11767225	+
Ro05_G14831	*RoCSE2*	1.60E-168	Ro05	8388911	8389882	-
Ro03_G06463	*RoCSE3*	3.21E-92	Ro03	41371272	41372237	+
Ro02_G01341	*RoCSE4*	2.13E-64	Ro02	7555107	7562042	+
Ro07_G22034	*RoCSE5*	3.01E-60	Ro07	33308609	33309595	+
Ro04_G02691	*RoCSE6*	4.61E-60	Ro04	4776071	4780782	-
Ro03_G26661	*RoCSE7*	4.83E-60	Ro03	23741694	23743524	-
Ro01_G00832	*RoCSE8*	1.64E-58	Ro01	2927160	2928092	+
Ro04_G36219	*RoCSE9*	4.07E-57	Ro04	4772872	4775565	-
Ro07_G18799	*RoCSE10*	1.24E-56	Ro07	34342592	34356142	+
Ro05_G03655	*RoCSE11*	1.29E-40	Ro05	424656	429569	-
Ro02_G00679	*RoCSE12*	5.82E-30	Ro02	2895098	2897978	-
Ro06_G04873	*RoCSE13*	0.001	Ro06	23003354	23007264	-
Ro04_G02559	*RoCSE14*	0.002	Ro04	3973434	3976314	+
Ro05_G13809	*RoCSE15*	0.003	Ro05	13203890	13209783	+
AT1G52760.1	*ATCSE1*	0	Chr1	19651325	19652743	+
AT1G11090.1	*ATCSE2*	1.79E-90	Chr1	3702608	3703779	+
AT3G62860.1	*ATCSE3*	1.79E-67	Chr3	23239446	23242251	-
AT2G47630.1	*ATCSE4*	1.11E-64	Chr2	19534503	19537014	+
AT5G14980.1	*ATCSE5*	2.83E-64	Chr5	4849600	4850583	+
AT3G55190.1	*ATCSE6*	4.96E-63	Chr3	20458007	20459890	+
AT2G39420.1	*ATCSE7*	9.88E-63	Chr2	16460364	16463061	+
AT3G62860.2	*ATCSE8*	1.63E-61	Chr3	23239264	23242274	-
AT5G16120.1	*ATCSE9*	5.78E-59	Chr5	5265939	5267979	+
AT5G16120.3	*ATCSE10*	5.78E-59	Chr5	5265473	5267954	+
AT2G47630.2	*ATCSE11*	1.12E-58	Chr2	19534503	19537014	+
AT5G16120.2	*ATCSE12*	1.21E-58	Chr5	5265633	5267916	+
AT5G16120.4	*ATCSE13*	1.43E-58	Chr5	5265617	5268078	+
AT2G47630.3	*ATCSE14*	1.96E-58	Chr2	19535143	19537014	+
AT1G77420.1	*ATCSE15*	5.47E-58	Chr1	29093499	29095716	+
AT2G39400.1	*ATCSE16*	3.82E-57	Chr2	16452612	16454801	+
AT5G19290.1	*ATCSE17*	3.36E-54	Chr5	6494055	6495284	+
AT3G55180.1	*ATCSE18*	6.53E-52	Chr3	20454903	20456682	+
AT5G11650.1	*ATCSE19*	6.55E-45	Chr5	3744980	3747125	+
AT1G73480.1	*ATCSE20*	2.80E-42	Chr1	27628939	27632806	+
AT1G18360.1	*ATCSE21*	1.26E-37	Chr1	6316695	6319427	-
AT2G39400.2	*ATCSE22*	1.46E-32	Chr2	16452612	16454801	+
AT5G11650.2	*ATCSE23*	1.82E-23	Chr5	3744980	3747149	+
AT2G19550.1	*ATCSE24*	3.02E-07	Chr2	8464533	8465531	-
AT3G47590.1	*ATCSE25*	4.03E-05	Chr3	17535759	17537530	-
AT5G38220.3	*ATCSE26*	5.13E-05	Chr5	15268827	15271386	+

AT5G38220.1	*ATCSE27*	5.13E-05	Chr5	15268868	15271353	+
AT1G13610.1	*ATCSE28*	0.001	Chr1	4663831	4666017	-
PCP011849.1	*PCCSE1*	2.05E-90	scaffold00368	36096	37366	+
PCP007413.1	*PCCSE2*	4.92E-65	scaffold00865	23689	26167	-
PCP015174.1	*PCCSE3*	4.98E-63	scaffold00530	135736	136870	+
PCP044524.1	*PCCSE4*	4.47E-62	scaffold00438	134353	137592	+
PCP027588.1	*PCCSE5*	4.98E-60	scaffold00158	58697	64117	-
PCP033740.1	*PCCSE6*	3.16E-59	scaffold00007	225900	228311	-
PCP044496.1	*PCCSE7*	1.64E-54	scaffold00388	47387	51504	+
PCP033156.1	*PCCSE8*	2.83E-53	scaffold00325	230224	233098	-
PCP045179.1	*PCCSE9*	3.29E-50	scaffold03738	2222	6678	+
PCP037709.1	*PCCSE10*	6.25E-46	scaffold138019	234	500	-
PCP027928.1	*PCCSE11*	1.06E-43	scaffold00498	127037	131727	-
PCP006868.1	*PCCSE12*	1.33E-42	scaffold00145	203696	206600	+
PCP025117.1	*PCCSE13*	2.29E-41	scaffold01196	66956	71990	+
PCP030138.1	*PCCSE14*	6.61E-36	scaffold09319	716	3220	+
PCP040027.1	*PCCSE15*	5.37E-35	scaffold16147	388	1648	-
PCP020858.1	*PCCSE16*	8.25E-14	scaffold00014	189066	193379	+
PCP005039.1	*PCCSE17*	8.75E-05	scaffold00004	783386	787490	+
PCP004967.1	*PCCSE18*	0.000642	scaffold00004	71986	74453	+
PCP001353.1	*PCCSE19*	0.001	scaffold02861	5956	8500	+
Pbr002315.3	*PbCSE1*	4.84E-109	Chr17	417048	418317	+
Pbr026316.1	*PbCSE2*	6.44E-65	scaffold424.0	38552	40101	-
Pbr026363.1	*PbCSE3*	6.44E-65	scaffold424.0	335722	337270	+
Pbr039057.1	*PbCSE4*	4.80E-64	scaffold828.0	51217	52209	-
Pbr032308.1	*PbCSE5*	5.88E-63	Chr7	10475405	10478354	+
Pbr026463.1	*PbCSE6*	6.61E-63	Chr7	9780086	9783240	-
Pbr003260.1	*PbCSE7*	9.80E-63	Chr11	90243	91971	+
Pbr042390.1	*PbCSE8*	2.43E-62	Chr3	27244839	27246437	-
Pbr038157.1	*PbCSE9*	2.76E-62	scaffold797.0	161366	165308	+
Pbr003259.1	*PbCSE10*	7.97E-60	Chr11	78090	79811	+
Pbr013306.1	*PbCSE11*	7.77E-58	Chr3	21021920	21025814	+
Pbr034764.1	*PbCSE12*	1.68E-54	Chr17	1718219	1722403	+
Pbr013307.2	*PbCSE13*	3.03E-52	Chr3	21019023	21021277	+
Pbr032606.2	*PbCSE14*	2.97E-50	Chr9	3640437	3644791	+
Pbr028331.1	*PbCSE15*	5.67E-45	Chr6	2620950	2625674	+
Pbr038811.1	*PbCSE16*	1.34E-43	Chr14	16893331	16898472	+
Pbr008780.1	*PbCSE17*	2.15E-42	Chr15	8768139	8770533	-
Pbr002316.1	*PbCSE18*	2.24E-40	Chr17	418552	419030	+
Pbr000313.1	*PbCSE19*	5.59E-14	Chr5	26316512	26317545	+
Pbr041464.1	*PbCSE20*	0.000102	Chr2	18579530	18584136	+
Pbr027746.2	*PbCSE21*	0.000329	Chr2	16457823	16459791	-
Pbr015620.1	*PbCSE22*	0.003	Chr15	14831830	14833767	+
Pbr042754.1	*PbCSE23*	0.004	Chr15	6452652	6454811	+

Pbr041469.2	*PbCSE24*	0.006	Chr2	18552536	18554396	+
FvH4_6g30610.1	*FvCSE1*	0	Fvb6	23705862	23706851	+
FvH4_3g04590.1	*FvCSE2*	5.16E-94	Fvb3	2627189	2628148	-
FvH4_2g25730.1	*FvCSE3*	5.01E-71	Fvb2	20784936	20788630	-
FvH4_1g06460.1	*FvCSE4*	2.30E-65	Fvb1	3410244	3411131	+
FvH4_3g25850.1	*FvCSE5*	6.85E-63	Fvb3	18696258	18700846	+
FvH4_7g20970.1	*FvCSE6*	7.23E-63	Fvb7	16931877	16934612	+
FvH4_7g19610.1	*FvCSE7*	1.44E-61	Fvb7	16116600	16117586	+
FvH4_4g27270.1	*FvCSE8*	7.29E-56	Fvb4	28329851	28332764	+
FvH4_4g27260.1	*FvCSE9*	5.27E-52	Fvb4	28326839	28329151	+
FvH4_2g35400.1	*FvCSE10*	1.08E-42	Fvb2	26111866	26114552	+
FvH4_5g17790.1	*FvCSE11*	1.56E-40	Fvb5	10193910	10199657	+
MD10G1300100	*MDCSE1*	4.88E-93	Chr10	38745228	38746256	+
MD03G1288300	*MDCSE2*	5.14E-70	Chr03	36701304	36707923	-
MD11G1309800	*MDCSE3*	6.17E-70	Chr11	42315614	42319994	+
MD07G1174800	*MDCSE4*	2.34E-64	Chr07	25347166	25348158	+
MD01G1108700	*MDCSE5*	8.68E-63	Chr01	22157195	22160070	+
MD01G1119700	*MDCSE6*	6.48E-62	Chr01	23347436	23351576	+
MD07G1188700	*MDCSE7*	7.29E-61	Chr07	26945274	26948322	+
MD16G1042000	*MDCSE8*	3.48E-59	Chr16	2958014	2960730	+
MD09G1252900	*MDCSE9*	3.88E-59	Chr09	32405681	32410472	+
MD16G1041900	*MDCSE10*	5.16E-57	Chr16	2949505	2957478	+
MD17G1244600	*MDCSE11*	4.05E-55	Chr17	29299235	29304001	+
MD06G1233800	*MDCSE12*	6.15E-45	Chr06	36502108	36507270	+
MD14G1240600	*MDCSE13*	3.57E-44	Chr14	31950828	31955830	+
MD15G1091700	*MDCSE14*	5.46E-43	Chr15	6363670	6366529	-
MD08G1112500	*MDCSE15*	9.56E-43	Chr08	9940108	9943040	-
MD05G1322100	*MDCSE16*	4.92E-36	Chr05	44952957	44953682	+
MD02G1199900	*MDCSE17*	8.14E-21	Chr02	19673208	19685871	+
MD13G1040700	*MDCSE18*	1.06E-17	Chr13	2793374	2794551	+
MD07G1114200	*MDCSE19*	9.30E-09	Chr07	13683967	13684221	-
MD15G1213300	*MDCSE20*	0.000565	Chr15	17118247	17121661	-
MD02G1086100	*MDCSE21*	0.000726	Chr02	6755952	6759379	-
Pm023999	*PmCSE1*	0	Pm7	9815109	9816062	+
Pm009952	*PmCSE2*	1.17E-92	Pm3	1803044	1804012	-
Pm020027	*PmCSE3*	9.56E-71	Pm6	814185	817561	-
Pm029072	*PmCSE4*	1.60E-59	scaffold205	1234119	1236150	-
Pm018723	*PmCSE5*	1.37E-58	Pm5	20230879	20232937	+
Pm029071	*PmCSE6*	9.91E-58	scaffold205	1230266	1233383	-
Pm029516	*PmCSE7*	1.18E-48	scaffold266	59987	68462	+
Pm016617	*PmCSE8*	1.99E-45	Pm5	3188067	3193518	+
Pm025280	*PmCSE9*	6.99E-45	Pm7	16912167	16917837	-
Pm005167	*PmCSE10*	4.19E-44	Pm2	9611910	9614332	+
Pm005170	*PmCSE11*	8.00E-44	Pm2	9617300	9620160	+

Pm018540	*PmCSE12*	1.01E-31	Pm5	19077991	19078641	+
Pm009567	*PmCSE13*	2.78E-16	Pm2	41863844	41865065	-
Prupe.5G109300.1.p	*PrCSE1*	0	Pp05	11444294	11446331	-
Prupe.4G040700.1.p	*PrCSE2*	7.19E-93	Pp04	1914947	1915966	-
Prupe.8G261500.1.p	*PrCSE3*	1.74E-69	Pp08	22077044	22081224	-
Prupe.1G312200.1.p	*PrCSE4*	1.11E-62	Pp01	30291520	30293997	-
Prupe.3G157400.1.p	*PrCSE5*	1.39E-60	Pp03	17594861	17600504	+
Prupe.2G213900.1.p	*PrCSE6*	1.46E-59	Pp02	24528305	24529492	+
Prupe.2G226400.1.p	*PrCSE7*	4.01E-58	Pp02	25287132	25290401	+
Prupe.1G312100.1.p	*PrCSE8*	3.71E-56	Pp01	30287567	30291123	-
Prupe.5G238400.1.p	*PrCSE9*	2.41E-44	Pp05	18014196	18021043	+
Prupe.1G446700.1.p	*PrCSE10*	3.10E-44	Pp01	37773754	37776527	-
Prupe.3G146300.1.p	*PrCSE11*	0.000867	Pp03	16001002	16007939	-
Potri.003G059200.1.p	*PoCSE1*	0	Chr03	8590854	8593871	-
Potri.001G175000.1.p	*PoCSE2*	0	Chr01	15048240	15050382	+
Potri.011G046500.1.p	*PoCSE3*	3.16E-88	Chr11	3644103	3645413	-
Potri.014G129000.1.p	*PoCSE4*	7.99E-69	Chr14	8649670	8654120	+
Potri.002G204200.1.p	*PoCSE5*	1.33E-67	Chr02	16884483	16888166	+
Potri.017G114800.4.p	*PoCSE6*	5.13E-63	Chr17	12241178	12246057	+
Potri.017G114800.1.p	*PoCSE7*	1.90E-62	Chr17	12241178	12246057	+
Potri.010G212200.1.p	*PoCSE8*	7.32E-62	Chr10	20049814	20052628	-
Potri.014G129000.3.p	*PoCSE9*	3.58E-61	Chr14	8649670	8654120	+
Potri.001G466700.1.p	*PoCSE10*	4.96E-60	Chr01	49157554	49158555	-
Potri.008G040000.1.p	*PoCSE11*	2.03E-58	Chr08	2237952	2239498	+
Potri.008G049000.1.p	*PoCSE12*	2.87E-58	Chr08	2864262	2867016	+
Potri.010G222300.1.p	*PoCSE13*	4.67E-57	Chr10	20700091	20701440	-
Potri.010G124000.1.p	*PoCSE14*	7.03E-57	Chr10	14172963	14177362	+
Potri.018G045800.1.p	*PoCSE15*	3.48E-45	Chr18	3910067	3913772	+
Potri.006G237200.2.p	*PoCSE16*	1.21E-43	Chr06	23907564	23911467	-
Potri.012G041800.7.p	*PoCSE17*	3.00E-43	Chr12	3721495	3727269	-
Potri.012G041800.1.p	*PoCSE18*	7.11E-43	Chr12	3720119	3727203	-
Potri.015G031900.4.p	*PoCSE19*	4.79E-41	Chr15	2455509	2461298	+
Potri.011G164300.2.p	*PoCSE20*	2.82E-34	Chr11	18125120	18125483	-
Potri.012G041800.2.p	*PoCSE21*	2.19E-17	Chr12	3756797	3763917	-
Potri.004G099700.1.p	*PoCSE22*	1.29E-10	Chr04	8612851	8612982	-
Potri.004G097300.1.p	*PoCSE23*	0.000174	Chr04	8612851	8614187	+
Pav_sc0000869.1_g590.1.mk	*PaCSE1*	5.49E-170	PAV_r1.0chr5	8962318	8963124	-
Pav_sc0000130.1_g1060.1.mk	*PaCSE2*	5.83E-69	PAV_r1.0chr8	20259161	20263255	-
Pav_sc0000554.1_g2080.1.mk	*PaCSE3*	3.91E-60	PAV_r1.0chr2	19566233	19568808	+
Pav_sc0000084.1_g340.1.mk	*PaCSE4*	4.29E-59	PAV_r1.0chr2	18603551	18607387	+
Pav_sc0001051.1_g150.1.mk	*PaCSE5*	9.19E-57	PAV_r1.0chr1	24617603	24623572	-
Pav_sc0002009.1_g230.1.mk	*PaCSE6*	7.20E-47	PAV_r1.0chr4	2369295	2369849	-
Pav_sc0000800.1_g640.1.mk	*PaCSE7*	1.35E-42	PAV_r1.0chr1	32919951	32927557	-
Pav_sc0001488.1_g300.1.mk	*PaCSE8*	7.40E-41	PAV_r1.0chr3	9157751	9167363	-

Pav_co4058231.1_g010.1.mk	*PaCSE9*	2.62E-35	PAV_r1.0chr0	1.68E+08	1.68E+08	+
Pav_sc0000103.1_g1170.1.mk	*PaCSE10*	7.32E-13	PAV_r1.0chr5	16512290	16518121	+

Table S3. The information about the *CSE* genes in ten species from the family evolution tree.
